# 2757. Activity of meropenem-vaborbactam tested against *Burkholderia* species isolates

**DOI:** 10.1093/ofid/ofad500.2368

**Published:** 2023-11-27

**Authors:** Mariana Castanheira, Dee Shortridge, Kelley Fedler, Cecilia G Carvalhaes

**Affiliations:** JMI Laboratories, North Liberty, Iowa; JMI Laboratories, North Liberty, Iowa; JMI Laboratories, North Liberty, Iowa; JMI Laboratories, North Liberty, Iowa

## Abstract

**Background:**

*Burkholderia* species can cause chronic and often severe respiratory tract infections in cystic fibrosis (CF) patients. The treatment of infections *Burkholderia* isolates is complicated by low cell permeability to most antimicrobial agents, presence of beta-lactamases and expression of efflux systems. We evaluated the activity of meropenem-vaborbactam (MEV) and comparator agents tested against *Burkholderia* spp. isolates collected during a surveillance study.

**Methods:**

A total of 328 *Burkholderia* spp. isolates were received as part of a global surveillance program from 2014 to 2022. Isolates were identified using MALDI-TOF MS and included *B. cenocepacia* (11), *B. cepacia* species complex (BCC; 268), *B. gladioli* (28) and *B. multivorans* (21). Isolates were susceptibility (S) tested by reference broth microdilution methods and CLSI interpretative criteria was applied for comparator agents for BCC isolates. Meropenem (MEM) alone breakpoints published by CLSI for BCC or EUCAST for *Pseudomonas aeruginosa* breakpoints were applied for MEV for comparison purposes.

**Results:**

MEV (MIC_50/90_, 1/2 mg/L) inhibited 95.1% of the BCC isolates at ≤4 mg/L and 97.0% at ≤8 mg/L. Trimethoprim-sulfamethoxazole (T/S), MEM and ceftazidime (CAZ) were active against 87.7%, 86.9% and 84.3% of the BCC isolates, respectively and levofloxacin and minocycline inhibited 63.4% and 82.5% of BCC isolates, respectively. Against *B. multivorans* (21), MEV (MIC_50/90_, 0.5/2 mg/L) inhibited 95.2% of the isolates at ≤4 mg/L or ≤8 mg/L while MEM inhibited 71.4% of the isolates at ≤4 mg/L and 95.2% of the isolates at ≤8 mg/L. T/S and CAZ were active against 71.4% and 81.0% of the *B. multivorans* isolates. T/S MEV, MEM and CAZ were active against all 11 *B. cenocepacia* isolates. MEM and MEV (MIC_50/90_, 0.5/2 mg/L) exhibited the same activity against 28 *B. gladioli* isolates.

**Conclusion:**

Chronic pulmonary infections caused by *Burkholderia* spp. isolates can lead to respiratory failure which is the primary cause of mortality and morbidity in CF patients. Despite the elevated susceptibility rates for the therapies of choice for these isolates, T/S, CAZ and MEM resistant isolates have been reported. MEV demonstrated good activity against BCC isolates that are commonly recovered from CF patients.

**Disclosures:**

**Mariana Castanheira, PhD**, AbbVie: Grant/Research Support|Basilea: Grant/Research Support|bioMerieux: Grant/Research Support|Cipla: Grant/Research Support|CorMedix: Grant/Research Support|Entasis: Grant/Research Support|Melinta: Grant/Research Support|Paratek: Grant/Research Support|Pfizer: Grant/Research Support|Shionogi: Grant/Research Support **Dee Shortridge, PhD**, Melinta: Grant/Research Support|Shionogi: Grant/Research Support **Kelley Fedler, BS**, Melinta: Grant/Research Support|Paratek: Grant/Research Support **Cecilia G. Carvalhaes, MD, PhD**, AbbVie: Grant/Research Support|bioMerieux: Grant/Research Support|Cipla: Grant/Research Support|CorMedix: Grant/Research Support|Melinta: Grant/Research Support|Pfizer: Grant/Research Support

